# Effective sociodemographic population assessment of elusive species in ecology and conservation management

**DOI:** 10.1002/ece3.670

**Published:** 2013-07-30

**Authors:** Josephine S Head, Christophe Boesch, Martha M Robbins, Luisa I Rabanal, Loïc Makaga, Hjalmar S Kühl

**Affiliations:** Department of Primatology, Max Planck Institute for Evolutionary AnthropologyDeutscher Platz 6, 04103, Leipzig, Germany

**Keywords:** Camera trapping, chimpanzee, conservation management, density, elephant, gorilla, multiple species monitoring, spatially explicit capture–recapture

## Abstract

Wildlife managers are urgently searching for improved sociodemographic population assessment methods to evaluate the effectiveness of implemented conservation activities. These need to be inexpensive, appropriate for a wide spectrum of species and straightforward to apply by local staff members with minimal training. Furthermore, conservation management would benefit from single approaches which cover many aspects of population assessment beyond only density estimates, to include for instance social and demographic structure, movement patterns, or species interactions. Remote camera traps have traditionally been used to measure species richness. Currently, there is a rapid move toward using remote camera trapping in density estimation, community ecology, and conservation management. Here, we demonstrate such comprehensive population assessment by linking remote video trapping, spatially explicit capture–recapture (SECR) techniques, and other methods. We apply it to three species: chimpanzees *Pan troglodytes troglodytes*, gorillas *Gorilla gorilla gorilla,* and forest elephants *Loxodonta cyclotis* in Loango National Park, Gabon. All three species exhibited considerable heterogeneity in capture probability at the sex or group level and density was estimated at 1.72, 1.2, and 1.37 individuals per km^2^ and male to female sex ratios were 1:2.1, 1:3.2, and 1:2 for chimpanzees, gorillas, and elephants, respectively. Association patterns revealed four, eight, and 18 independent social groups of chimpanzees, gorillas, and elephants, respectively: key information for both conservation management and studies on the species' ecology. Additionally, there was evidence of resident and nonresident elephants within the study area and intersexual variation in home range size among elephants but not chimpanzees. Our study highlights the potential of combining camera trapping and SECR methods in conducting detailed population assessments that go far beyond documenting species diversity patterns or estimating single species population size. Our study design is widely applicable to other species and spatial scales, and moderately trained staff members can collect and process the required data. Furthermore, assessments using the same method can be extended to include several other ecological, behavioral, and demographic aspects: fission and fusion dynamics and intergroup transfers, birth and mortality rates, species interactions, and ranging patterns.

## Introduction

Accurate animal population assessments are crucial for determining conservation priorities and measuring the success of implemented management strategies (Nichols and Williams 2006). Extensive population assessments which go well beyond estimates of density and population size are often necessary for the following: to evaluate the general impact of logging or mining on resident wildlife (Wrege et al. 2010), to assess the impact of hunting on species demography (Caro et al. 2009), or to evaluate the effectiveness of particular interventions and management activities (Campbell et al. 2011; Robbins et al. 2011; Tranquilli et al. 2012); and resulting consequences for biodiversity. Wildlife managers are therefore urgently searching for improved methods which are widely applicable, inexpensive, and precise, and which provide sufficient information on the population status of multiple species. These methods need to be applied by protected area local staff members because capacity building is central to the long-term success of conservation management (Rodriguez et al. 2006). Furthermore, a single approach to monitoring and conservation-oriented science and management would substantially improve conservation efficiency (Nichols and Williams 2006).

Past approaches to population assessment of elusive species have included line transect sampling (Buckland et al. 2001; Marques et al. 2001; Morgan et al. 2006), genetic sampling (Arandjelovic et al. 2010, 2011) audio recordings (Barlow and Taylor 2005; Wrege et al. 2010) or direct observations (White et al. 1993; Kindberg et al. 2009). Although widely applied and efficient for density estimation, several of these approaches have certain limitations when applied to comprehensive population assessments which also require information on sociodemographic parameters: they are applicable only to a certain group of species or specific question, are unable to monitor movements of individuals, or are reliant on indirect signs; all of which increase estimate uncertainty without providing sociodemographic information. Additionally, some methods are expensive and labor intensive (Arandjelovic et al. 2010) or lack precision for measuring small-scale changes (Plumptre 2000), while parameters such as sign decay or production rates necessary for analysis are often site specific (Kuehl et al. 2007; Kühl et al. 2008).

The increasing availability of remote camera traps combined with rapid developments in wildlife statistics has enabled significant progression of monitoring efficiency in the last 10 years. Camera traps are quickly gaining popularity among researchers and conservationists, and enable long-term spatio-temporal monitoring of specific individuals or populations over many years. To date, they have primarily been used to measure species richness or estimate abundance using capture–recapture (C-R) in single species studies (Karanth and Nichols 1998; O'Connell et al. 2011). However, increasingly they are being employed to monitor other aspects of ecology and behavior including activity patterns (Harmsen et al. 2011), feeding ecology (Leuchtenberger et al. 2012), interspecific competition (Head et al. 2012), and disease screening (Oleaga et al. 2011).

There are numerous statistical models for estimating abundance using noninvasive and nonspatial C-R techniques (Otis et al. 1978; White and Burnham 1999; Miller et al. 2005). Density is then often inferred by dividing the C-R estimates of abundance by the size of the area sampled. However, this can lead to inflated density estimates because the effective sample area is typically smaller than the area occupied by the individuals identified (Obbard et al. 2010). Individuals which are detected toward the outer limit of the area sampled are therefore also likely to range outside, and this effect leads to overestimates of density.

In order to provide unbiased density estimates, C-R methods must therefore take into account the effect of temporary emigration of individuals from the sampling area. Recent developments of spatially explicit capture–recapture (SECR) techniques have overcome this limitation of traditional C-R models and enabled robust density estimates (Borchers and Efford 2008; Efford et al. 2009; Royle et al. 2009; Gardner et al. 2010; Chandler and Royle 2011). SECR methods determine the activity centers of individuals. The estimated number of activity centers across a precisely defined polygon then gives both density and abundance (Efford et al. 2009). Comparative studies have shown that using this method results in lower density estimates than nonspatial methods (Noss et al. 2012). SECR models are also able to incorporate other important sources of variation such as intersexual or interindividual variability in ranging patterns and capture probability (Sollmann et al. 2011); but like traditional C-R models, they assume a closed population.

The development of SECR methods has gained momentum over the last few years and a variety of methods exist to date. These methods often have ready to use software packages freely available, including likelihood-based SECR (Efford 2012, http://www.otago.ac.nz/density; http://cran.r-project.org/web/packages/secr/index.html) and Bayesian hierarchical SECR (Royle et al. 2009, http://cran.r-project.org/web/packages/SPACECAP/SPACECAP.pdf).

Like many habitats on earth, the landscape of the African rainforests is rapidly changing (Blake et al. 2007; Junker et al. 2012), and both protected area managers and wildlife biologists are in urgent need of improved population assessment methods. The overall aim of this study was to develop an easy-to-use approach for conducting an extensive multispecies population assessment for conservation management which could be widely applied across rainforest habitats. We selected three mammal species from Loango National Park, Gabon for this study: endangered chimpanzees *Pan troglodytes troglodytes*, western gorillas *Gorilla gorilla gorilla,* and forest elephants *Loxodonta cyclotis*. They are all elusive and live in densely forested habitats, making them ideal candidates for such a study. Specific objectives were to estimate abundance, density, home range size, and examine the social and demographic structure of these three sympatric species. In order to investigate if remote camera traps could answer questions about the differing social structure of chimpanzees, gorillas, and elephants, we examined group size and distribution, age–sex structure, intersexual variation in home range size, and the existence of both resident and nonresident elephants within the population. The availability of information from other sources allowed comparisons and partial validation of our results. Finally, we wanted to investigate the ability of untrained observers in subjective identification of individual animals in order to confirm the suitability of the method for protected area staff members.

Our findings indicate that the combination of remote camera trapping and SECR techniques provides a very powerful, widely applicable approach for extensive population assessments that can be applied by park staff members trained in camera maintenance and individual identification.

## Material and Methods

### Field methods

#### Study site

**Figure 1:**
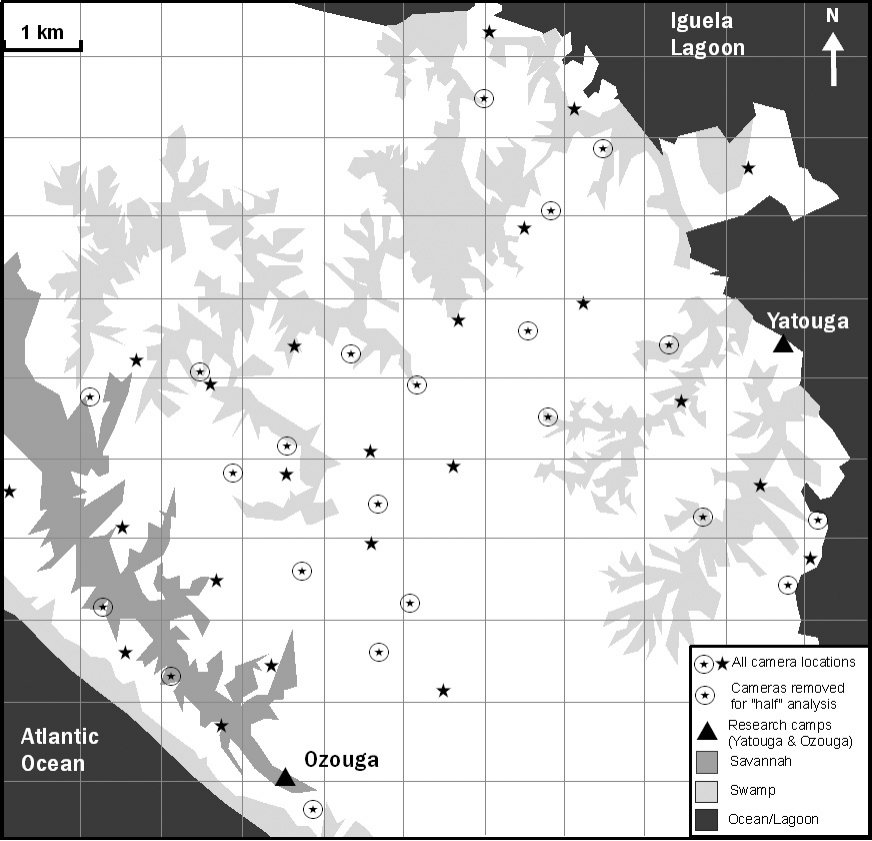
Study area and camera trap distribution showing the full array of cameras, and cameras removed from the analysis when running “half models” which included every second camera in the grid cells.

The study site of the Loango ape project (Fig. 1) is located in Loango National Park, Gabon (2º04′S, 9º33′E), covering 160 km² on a peninsula bordered to the west by the Atlantic Ocean and to the east by a large lagoon. Habitat type was heterogeneous (including mature, secondary, coastal, and swamp forest), mean annual rainfall (collected daily) was 2215 mm, and the mean minimum and maximum temperatures per day were 22.9°C and 27.2°C, respectively (Head et al. 2011).

#### Remote camera traps

Over a 20-month period between April 2009 and November 2010, we used between seven and 45 remote camera traps (see Appendix S1) set up in a systematically defined grid of 1 km^2^ squares across the 60 km^2^ study area (Fig. 1). One assumption of the SECR model is that demographic closure exists within a population. An extended data collection period such as ours would be expected to violate this assumption. However, chimpanzees, gorillas, and elephants are all very slowly reproducing species (4–6 year interbirth intervals) with long life histories, reducing individual turnover. We discuss the issue of prolonged sampling periods and potential violation of population closure in the discussion section.

Cameras were placed systematically with one per grid square, but within the square they were placed optimally to increase capture probability. The distribution of the cameras ensured that all individuals within the study area were exposed to cameras, based on each species known minimum home range size (Chapman and Wrangham 1993; Cipolletta 2003; Blake et al. 2008). Cameras were located in neutral areas such as animal trails and natural bridges that were equally accessible to and regularly used by all species (J. Head, pers. obs.). Cameras were not moved during the study to avoid biasing the data. Due to limitations on camera availability, there were some grid cells without cameras. Motion detectors in the cameras were programed to trigger immediately when movement was detected and were active for 24 h a day. Cameras were checked once every 2 weeks by two researchers, and 5 days were needed to check 45 cameras.

### Analytical methods

We used camera trap data and maximum likelihood-based SECR techniques (Efford 2012, SECR 2.3.2 http://www.otago.ac.nz/density) to estimate abundance, density, and home range size of chimpanzees, gorillas, and elephants. We also derived demographic group composition and assessed association patterns to infer group social structure. Furthermore, we measured the agreement in individual identification between the principal investigator (J. Head) and several trained and untrained observers using Cohen's Kappa coefficient.

#### Capture events and discrimination of individuals

Identification of chimpanzees, gorillas, and elephants was restricted to weaned individuals estimated to be >6 years old (determined by long-term observation of habituated chimpanzees [C. Boesch: Boesch and Boesch-Achermann 2000], gorillas [M. Robbins: Robbins et al. 2001], and elephants [A. Turkalo, unpubl. data] of known age). This was because the nest count and genetic methods used for chimpanzees and gorillas to which we compared our estimates excluded unweaned individuals since they are nonnest builders and produce very small feces, respectively. There were 1045, 471, and 2237 visits of chimpanzees, gorillas, and elephants, respectively, at remote camera trap locations. Of these, 439 (42%), 103 (22%), and 963 (43%) positive identifications were possible for the three species, respectively. Unique individuals were identified through a combination of facial and body characteristics. These characteristics are widely used and have been shown to lead to very high rates of correct identification. They include shape and coloration of ears, nose, face, and body (Fossey 1983; Goodall 1988), in addition to body scars or disfigurements (see Appendix S2). Elephants were additionally identified using tusk and tail dimensions (Goswami et al. 2011). Because body scars, size, and coloration can change over time, we only considered captures in which multiple (minimum of three) features of an individual were observed. Due to the large amount of footage obtained for the elephants, we only analyzed data from 24 cameras.

#### Interobserver reliability

All individual discrimination that was used for the SECR analysis was done by one experienced observer (J. Head). The principal investigator spent 6 years at the field site and observed many of the individuals directly. We could therefore assume a negligible misidentification rate. Additionally, we wanted to examine how well differently trained people would be able to identify individuals. We therefore compared the rate of agreement in identification between the principal investigator and several trained and untrained individuals by conducting interobserver reliability testing on all three species (Appendix S3). We tested two observers who had experience working with or observing the subject species, but no experience working with video footage for individual recognition; two Gabonese field assistants from the Loango ape project who had experience working with apes and camera traps; two Gabonese ecoguards from Loango National Park who had experience working in the forest, but not working with the subject species or camera traps; and finally two western observers who had no experience working with apes, elephants, or camera traps.

Participants studied independent pairs of video images with accompanying still shot photos (*N* = 40 pairs of chimpanzees and elephants, *N* = 24 pairs of gorillas) of their subject species and decided if the pairs were the same or different individuals. Interobserver reliability was measured using Cohen's Kappa coefficient (Cohen 1960) (see Appendix S3 for detailed methods) and differences in agreement between participant categories were compared using Wilcoxon signed-ranks matched-pairs tests (Siegel and Castellan 1988) applied to averages per participant category and age–sex class. Interspecies differences were tested using Friedman tests on scores per participant and species, followed by Wilcoxon tests for pairwise comparisons between species.

#### Estimating density

Density estimates were calculated in R (version 2.15.0; R development core team 2012) using the maximum likelihood-based SECR 2.3.2 package (Efford 2012) that was developed to overcome the limitations of traditional C-R methods which are not appropriate for density estimates using either camera traps or genetic methods (Efford et al. 2009; Sollmann et al. 2011). Similar to other likelihood-based capture–recapture models, SECR is a mixture model whereby the mixture pertains to the spatial distribution of each animal (Borchers and Efford 2008). However, the important difference between SECR and other likelihood-based C-R models is that the SECR model takes explicit account of temporary emigration of individuals from the sampling area (Borchers and Efford 2008).

Another approach to SECR models has also been developed more recently which uses a Bayesian hierarchical framework (Royle et al. 2009). Bayesian inferences are useful for arbitrary sample sizes and thus also ideal for small data sets (Gardner et al. 2010); however, the user-friendly SPACECAP R package for Bayesian SECR analysis was not yet available at the time of our study.

The SECR package estimates sampling area by suggesting a buffer width around the outermost cameras for each species (based on a habitat mask created over camera and capture locations of each individual) and after removing nonhabitat (sea and lagoon) from the buffer it resulted in an effective sampling area of 129 km^2^, 160 km^2^, and 146 km^2^ for chimpanzees, gorillas, and elephants, respectively. We assumed a half normal model with binomial distribution, and the capture data were split into 20 “trapping occasions” of 30 days each. Days when a camera was not filming due to not being installed or because of technical problems (such as a full memory card) were controlled for in the analysis and species-specific camera coverage (see Head et al. 2012), and sex of individuals were included as covariates in the model.

We ran a null model (null) which assumed capture probability was constant across all individuals, a second model (sex) which accounted for variation in density, capture probability, and home range size between males and females, and a third two-class finite mixture model (sexhet) which included additional heterogeneity in capture probability and home range size between individuals as well as between sexes. Output from the second model using the SECR package indicated that using a Poisson distribution was more appropriate than a binomial distribution for the gorilla and elephant data set, and we therefore used a Poisson distribution in all models for these two species; and a binomial distribution for chimpanzees. Because gorillas exhibit stable group structure (Robbins et al. 2004), home range size between group males and females should be equal. We therefore additionally ran the second and third models described above again but this time accounting for variation between solitary males and group individuals rather than between males and females (hereafter referred to as “group” and “grouphet”).

We compared the three models for chimpanzees and elephants and the six models for gorillas using the Akaike Information Criterion (AIC), and all estimates for each species were made using the model that fit best to the data. Abundance was calculated from the density estimates using the “region.N” function of the SECR package, and in all analysis we used 95% confidence intervals (Sollmann et al. 2011).

In order to measure the effect of the number of cameras (i.e., sample size) on the precision of the analysis, we reran all the models described above but excluding data from every second camera in the grid (Fig. 1). This resulted in a sample size of 234, 73, and 552 positive identifications from 541, 286, and 1308 visits for chimpanzees, gorillas, and elephants, respectively; again, we compared these “half models” using the AIC.

#### Assigning group membership and inferring social structure

Individuals captured together during the same video trigger or within 15 min of other individuals on the same camera were considered part of the same group or community. Furthermore, individuals that were not captured together, but which were both captured independently with a third individual in common, were considered part of the same group (Arandjelovic et al. 2010). For example, if A was captured with B, and B with C, then A and C were assumed to share group identity. Because gorilla groups are stable we were additionally able to infer minimum group size and composition through identifying only adult males and then assigning age/sex to every individual seen in a video clip with each adult male (Figs. S1–S3). In order to investigate the presence of resident and nonresident elephants within the study area, we measured residency rates during the 12-month period with most intensive camera usage (November 2009–November 2010). Data were split into 26 biweekly periods and presence/absence of each individual recorded. Residency was calculated as the average waiting time between capture events for each individual. Individuals with a waiting time ≤5 biweeks were considered resident and individuals ≥15 biweeks considered nonresident.

#### Estimating home range size

Mean species home range size was calculated using the “circular.r” function from the SECR package with 95% confidence intervals.

Additionally, approximate home range (AHR) sizes of groups and individuals were calculated using trap locations of all groups or individuals in ESRI® ArcMap™ 9.2 (Redlands, CA) using the minimum convex polygon (MCP) tool.

To measure the effect of sex on AHR of elephants, we ran a generalized linear mixed model (GLMM; Baayen 2008) which included sex, total number of observations and their interaction as fixed effects, and group identity as a random effect. We initially included random slopes of the effect of sex within groups into the model (assuming that sex differences could randomly vary between groups) but removed it since it appeared insignificant (*χ*^2^=0, df = 1, *P* = 1). Prior to fitting the models, we log-transformed and then z-transformed total number of observations (to a mean of zero and a standard deviation of one), and only individuals captured on at least two occasions were included in the analysis. The GLMM was fitted assuming normally distributed and homogeneous error and using the function lmer from the R-package lme4 (Bates and Maechler 2010) and reliable *P*-values for the individual terms in the model were achieved using Markov chain Monte Carlo (MCMC) sampling and the function “pvals.fnc” of the R-package “languageR” (Baayen 2008). We checked for the assumptions of normally distributed and homogeneous residuals by visual inspection of residuals plotted against predicted values and had no indications that these assumptions were violated after AHR was log-transformed.

**Figure 2:**
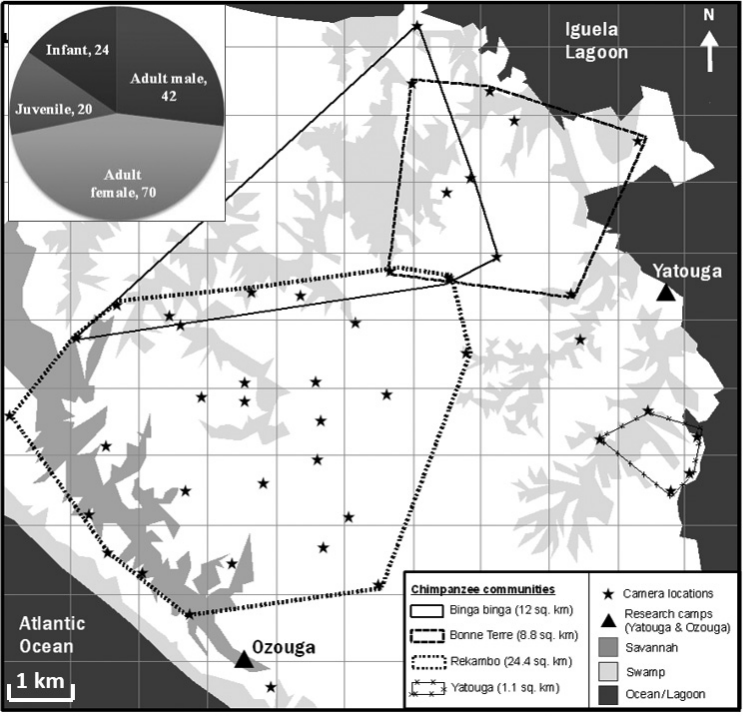
Map showing chimpanzee home ranges, minimum home range size, and camera distribution. Pie chart shows the age/sex structure of the population.

Because chimpanzee communities exhibit fission-fusion and because individuals from communities on the periphery of the sampling grid were often sampled only once, we used a subset of the data which included only individuals from the Rekambo community (Fig. 2) to measure the effect of sex on AHR of chimpanzees. Juveniles were excluded from the analysis because they did not move independently from their mothers, but all other community members were included irrespective of number of observations. In order to maximize sample size, we included 19 additional months of identification data from the Rekambo community collected on nine cameras between November 2010 and June 2012, which resulted in an additional 116 positive identifications. We ran a general linear model (LM) assuming normally distributed and homogeneous residuals and using the same model as the GLMM described above but excluding group identification as a random factor. We z-transformed total number of observations and log-transformed AHR.

To test specifically the effect of sex on home range size, we compared the full models (both GLMM and LM) to null models which excluded sex and its interaction with the number of observations. This comparison was done using a likelihood ratio test for elephants (Dobson 2002) and an *F*-test for chimpanzees. We did not test for the effect of sex on home range size of gorillas because males and females live in stable groups.

#### Evaluation of method

In order to evaluate the strength of the SECR method and allow direct comparisons with nonspatial methods previously used on the same population of chimpanzees and gorillas, we estimated abundance using the two innate rates model (TIRM) implemented in Capwire (for details see Arandjelovic et al. 2010). The precision of the elephant estimate was compared to previous genetic and dung count studies carried out in similar habitat in the equatorial rainforests of Kakum National Park, Ghana (Eggert et al. 2003), which also used nonspatial analytical methods. Estimate precision was measured as per Arandjelovic et al. (2010) as the entire width of the 95% confidence intervals divided by the estimate itself, and density was measured by dividing the abundance estimate by the area sampled.

Additionally, because our long data collection period potentially violated the demographic closure assumptions of the SECR model, we used home range size data from known individuals to look for evidence of possible violation in estimates among the three species. In order to evaluate the SECR method for estimating home range size of chimpanzees, gorillas, and elephants, we used the known home ranges of one chimpanzee community (Rekambo) and one gorilla group (Atananga) both under habituation within the study area. Home ranges of Rekambo and Atananga were measured by placing MCP's around all GPS locations where group members were either directly observed (*N* = 840 and 307 for chimpanzees and gorillas, respectively) or followed (*N* = 68 km and 260 km for chimpanzees and gorillas, respectively) during a 3-year period between 2009 and 2011 (for details on observation and follow definitions see Head et al. 2011) and home range sizes of Rekambo and Atananga were 36 km^2^ and 59 km^2^, respectively. Data on elephant ranging in Loango came from a radio collaring study by Blake et al. (2008), and mean home range size was 76 km^2^ (range = 26–158 km^2^).

We further looked for evidence that we may have violated the population closure assumptions of the SECR method with the duration of our study period using density data from a known population. We used direct observation contact data from the Rekambo chimpanzee community over the same 3-year period as above, where the total number of unique weaned individuals identified was 45. Dividing the number of weaned individuals by home range size based on direct observations resulted in a density of 1.25 individuals per km^2^ (hereafter referred to as “known density”). In order to obtain a density estimate for the Rekambo community, we ran the same SECR models as described above but with a subset of the data. We included all captures of Rekambo individuals, all cameras located within the known Rekambo home range and we considered areas outside of the home range as nonhabitat.

## Results

### Discrimination of individuals

There were 123, 52, and 139 unique individual chimpanzees, gorillas, and elephants identified from the video camera trap footage. Capture rate per individual ranged from 1–15 for chimpanzees (mean = 3.5, SD = 3.1), 1–8 for gorillas (mean = 2, SD = 1.42), and 1–46 for elephants (mean = 6.8, SD = 7.4). There were 44 (36% of individuals), 27 (52% of individuals), and 27 (20% of individuals) chimpanzees, gorillas, and elephants that were sampled only once during the whole period.

### Interobserver reliability and method testing with different observers

Among all participants, Cohen's Kappa agreement coefficient was higher than expected by chance for all species and sexes, but there was variation between species and participant category (see Appendix S3). Experienced observers had significantly higher agreement than nonexperienced observers (Wilcoxon test: *T*^+^ = 21, *N* = 6, *P* = 0.031) and tended to have higher agreements than ecoguards (*T*^+^ = 15, *N* = 5 (1 tie), *P* = 0.063), but field assistants could not be tested because of too small sample size due to tied observations. Misidentification rates also differed between species (Friedman test: *χ*^2^ = 13, df = 2, *P* = 0.002). There were significantly fewer misidentifications among elephants than either gorillas or chimpanzees (both tests: *T*^+^ = 36, *N* = 8, *P* = 0.008), but no significant difference between gorillas and chimpanzees (*T*^+^ = 28, *N* = 8, *P* = 0.195).

### Estimating density

Across all species the top SECR models accounted for heterogeneity in both capture probability and home range size among certain groups of individuals (Table 1). The “sexhet” model was the best fit for determining chimpanzee and elephant density and estimated 1.72 (1.54–1.95) and 1.37 (1.25–1.54) individuals per km^2^ with a male to female sex ratio of 1:2.1 and 1:2, respectively. The “grouphet” model was the best fit for determining gorilla density and estimated 1.2 (0.93–1.68) individuals per km^2^ with a male to female sex ratio of 1:3.2.

**Table 1:** Chimpanzee, gorilla, elephant, and Rekambo chimpanzee results of the different models evaluated: “null,” “sex,” “sexhet,” “group,” and “grouphet.”

Species	Model	No. par	logLik	AIC	AICc	dAICc	AICwt
Chimpanzee	Null	3	−1975	3957	3957	62.6	0
Chimpanzee	Sex	6	−1961	3934	3935	40.9	0
**Chimpanzee**	**Sexhet**	**9**	−**1937**	**3893**	**3894**	**0.0**	**1**
Gorilla	Null	3	−537	1080	1080	19.7	0
Gorilla	Sex	6	−529	1070	1072	11.7	0
Gorilla	Sexhet	9	−525	1068	1073	12.3	0
Gorilla	Group	6	−525	1062	1064	3.6	0.14
**Gorilla**	**Grouphet**	**9**	−**519**	**1056**	**1060**	**0.0**	**0.86**
Elephant	Null	3	−3886	7778	7778	250.2	0
Elephant	Sex	6	−3878	7769	7770	242.6	0
**Elephant**	**Sexhet**	**9**	−**3754**	**7526**	**7528**	**0.0**	**1**
Rekambo ch	Null	3	−1015	2036	2036	1.7	0.22
Rekambo ch	Sex	6	−1011	2034	2036	1.3	0.27
**Rekambo ch**	**Sexhet**	**9**	−**1006**	**2030**	**2035**	**0.0**	**0.51**

Null, null model; sex, heterogeneity between sexes; sexhet, heterogeneity between sexes and individuals; group, heterogeneity between group and solitary individuals; grouphet, heterogeneity between group and solitary, and between all individuals. No.par, number of parameters; logLik, the log likelihood; AIC, Akaike Information Criterion; AICwt, AIC weight data. The best fit models per species according to the AIC are shown in bold.

The chimpanzee and elephant half models resulted in a higher density estimate and increased SE than the full models which included data from all cameras (Table 2), whereas the gorilla half model resulted in a lower density estimate with reduced SE.

**Table 2:** Results of the full (all camera traps) and half (every second camera trap in the grid) SECR density estimate models for chimpanzees, gorillas, and elephants in Loango NP, Gabon

	Full models		Half models	
		
	Density estimate	SE	Density estimate	SE
Chimpanzee				
Null	1.49 (1.35–1.65)	0.08	1.14 (0.9–1.43)	0.13
Sex	1.42 (1.32–1.54)	0.11	1.15 (1–1.35)	0.17
Sexhet	**1.72 (1.54–1.95)**	**0.21**	**2.27 (1.73–3.29)**	**0.76**
Gorilla				
Null	0.71 (0.51–0.98)	0.12	0.74 (0.5–1.09)	0.15
Sex	0.79 (0.66–0.99)	0.18	0.82 (0.65–1.09)	0.21
Sexhet	1.08 (0.84–1.56)	0.33	1.2 (0.89–1.86)	0.47
Group	0.71 (0.61–0.87)	0.13	**0.75 (0.62–0.94)**	**0.16**
Grouphet	**1.2 (0.93–1.68)**	**0.37**	1.32 (0.96–2.14)	0.57
Elephant				
Null	1.33 (1.12–1.58)	0.12	1.41 (1.17–1.71)	0.14
Sex	1.34 (1.21–1.51)	0.15	1.41 (1.26–1.6)	0.18
Sexhet	**1.37 (1.25–1.54)**	**0.16**	**1.95 (1.73–2.28)**	**0.29**

Null, null model; sex, heterogeneity between sexes; sexhet, heterogeneity between sexes and individuals; group, heterogeneity between group and solitary individuals; grouphet, heterogeneity between group and solitary, and between all individuals. Results in bold denote the top SECR full and half models for each species.

### Assigning group membership and inferring social structure

Eleven chimpanzees were captured alone and thus could not be assigned to a group. The remaining 112 individuals were assigned to four communities containing 45, 32, 13, and 22 individuals (Fig. 2, see Fig. S1).

**Figure 3:**
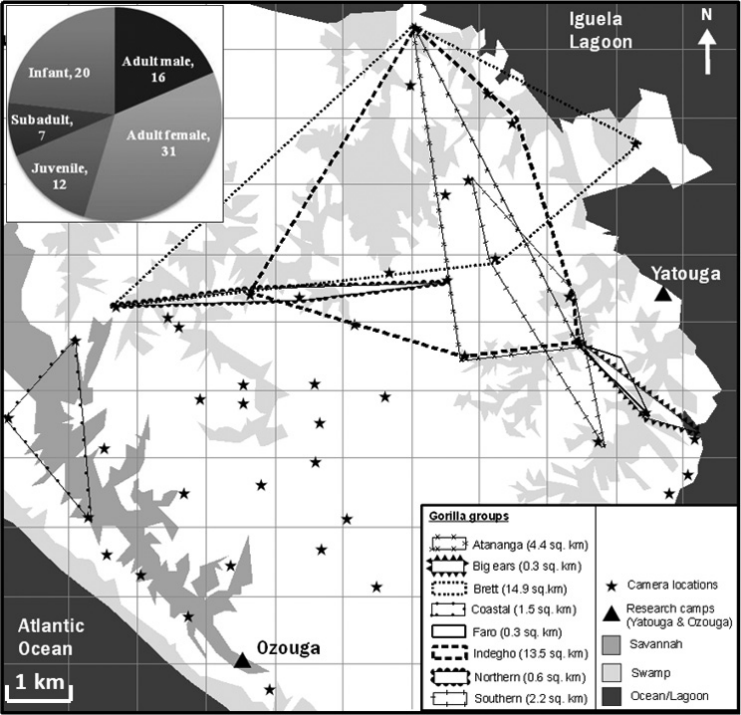
Map showing gorilla home ranges, minimum home range size, and camera distribution. Pie chart shows the age/sex structure of the population.

Gorillas were assigned to eight groups each with a silverback male and multiple females (Fig. 3), and there were eight solitary males and six females captured alone. Inference of minimum group size and composition through identification of group adult males showed that average number of adult females per group was 3.9 and average overall group size 9.5 individuals (see Fig. S2).

**Figure 4:**
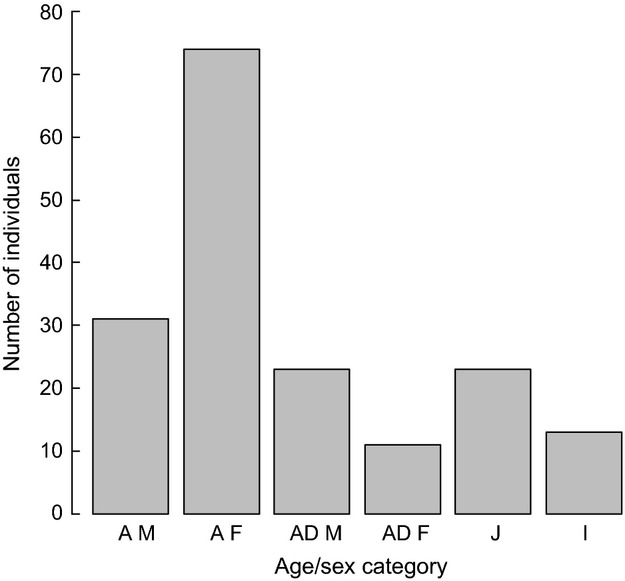
Age/sex structure of the elephant population in the study area. A, adult; AD, adolescent; J, juvenile; I, infant; M, male; F, female.

**Figure 5:**
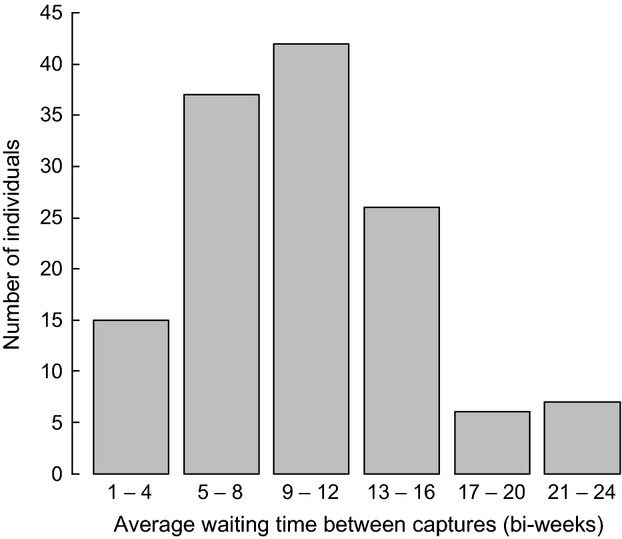
Residency scores of elephant individuals within the study area. Individual scores are the average number of biweeks between observations. Individuals with a waiting time ≤5 biweeks were considered resident and individuals with ≥15 considered nonresident.

Thirty-two adult female elephants and 36 adult male elephants were captured alone and thus could not be assigned to a group. The remaining individuals were assigned to 21 core groups composed of multiple adult females and their offspring (Fig. 4, see Fig. S3). Temporary fusing of two or three core groups was observed among females on four occasions. Fourteen adult males were observed in bachelor groups of two or three individuals on 18 occasions but were otherwise solitary, and there was variation in residency between individual elephants (Fig. 5), suggesting that there were both resident and nonresident elephants within the Loango population.

For elephants, a likelihood ratio test revealed that the full model fitted significantly better than the null model (GLMM: *χ*^2^ = 6.37, df = 2, *P* = 0.04). Furthermore, the interaction between sex and total number of observations was significant (*P*_MCMC_ = 0.01), indicating that the number of observations affected home range size of males and females differently (see Table S1). Inspection of the data suggested that males tended to have larger home ranges than females. For chimpanzees, there was no obvious effect of sex on home range size (full-null model comparison: *F*_2,32_ = 1.0, *P* = 0.37).

### Evaluation of SECR and comparison with other methods

The sexhet model was the best fit for determining chimpanzee density from the Rekambo community subset of data (Table 1) and resulted in an estimate of 1.34 (1.13–1.65) chimpanzees per km^2^, compared to the known density of 1.25 chimpanzees per km^2^ in this community. Average home range size for each species was estimated by the SECR models to be 41 km^2^, 50 km^2^, and 59 km^2^ for chimpanzees, gorillas, and elephants, respectively, compared to 36 km^2^, 59 km^2^, and 76 km^2^ (range = 26–158 km^2^; Blake et al. 2008) as measured by direct observations, respectively.

The TIRM method resulted in similar abundance scores but higher density estimates compared to the full SECR for chimpanzees and gorillas but not for elephants (Table 3). The chimpanzee density estimates using camera traps (both SECR and TIRM) were more precise than studies using either line transect or genetic sampling. The gorilla density estimate with SECR had lower precision than other methods, while with TIRM it was equal to line transects but less precise than genetic sampling (Table 3). The precision of the elephant estimates from camera traps with both the TIRM (2%) and SECR (22%) was higher than that estimated with genetic sampling (60%) or dung counts (80%) from Kakum, in Ghana (Eggert et al. 2003).

**Table 3:** Comparison of different methods for estimating densities of chimpanzees, gorillas, and elephants in Loango NP, Gabon

Species	Method	Abundance	Density estimate (per km^2^)	Precision^1^ (%)	Data collection period (months)	Sample size	Sampling area (km^2^)	Extraction/ID success (%)
Chimpanzee	Line transect^2^	unk^3^	unk^3^	52%^2^	4	322 km	101	n/a
	Genetic sampling^4^	283 (208–316)	2.14 (1.58–2.39)^5^	38%	48	444 feces	132	46%
	Camera trap (SECR)^6^	154 (144–169)	1.72 (1.54–1.95)	24%	20	956 ind.	129^7^	42%
	Camera trap (TIRM)^6^	155 (134–168)	2.58 (2.23–2.8)^8^	22%	20	956 ind.	60	42%
Gorilla	Line transect^2^	unk^3^	unk^3^	52%^2^	4	322 km	101	n/a
	Genetic sampling^2^	87–107	0.96 (0.86–1.96)^5^	33%	36	396 feces	101	82%
	Camera trap (SECR)^6^	82 (69–104)	1.2 (0.93–1.68)	63%	20	458 ind.	160^7^	22%
	Camera trap (TIRM)^6^	91 (62–109)	1.51 (1–1.8)^8^	52%	20	458 ind.	60	22%
Elephant	Camera trap (SECR)^6^	165 (156–180)	1.37 (1.25–1.54)	21%	20	2235 ind.	146^7^	43%
	Camera trap (TIRM)^6^	139 (138–141)	2.32 (2.3–2.35)^8^	2%	20	2235 ind.	60	43%

Includes previous line transect and genetic sampling (using TIRM method), and two camera trap analyses carried out in this study (SECR and TIRM).

1 Precision measured as per Arandjelovic et al. (2010, 2011) as entire width of 95% confidence intervals divided by the estimate itself.

2 Arandjelovic et al. 2010.

3 No abundance or density estimate could be calculated because data on nest decay and construction rate were absent.

4 Arandjelovic et al. 2011.

5 Estimates calculated by J. H. based on published abundance divided by area sampled.

6 This study.

7 Area sampled includes buffer area around camera traps as estimated by the SECR package.

8 Calculated as abundance divided by actual sampling area of camera traps (MCP: 60 km^2^ all species).

## Discussion

Our study provides an approach for making extensive sociodemographic population assessments across multiple species. It is straightforward to implement and wildlife managers can thus continuously monitor various levels of sociodemographic change in population characteristics that provide detailed information about conservation effectiveness. Our study also highlights the multipurpose use of camera traps that go well beyond evaluation of species richness and single species density estimation and have great potential for addressing many sociodemographic and ecological questions.

### Validity of density and home range estimates

SECR like other C-R methods are based on the assumption of population closure. Our study period was extended and may therefore have violated this assumption. However, the density estimate validation using data from a known chimpanzee community indicated that any potential violation was minimal. Additionally, comparing our results to traditional C-R and other methods validated the applicability of SECR for estimating both density and home range size of chimpanzees and elephants, while density estimates for gorillas using SECR were more difficult to interpret (Table 2). SECR methods are based on the assumption that individuals move independently and its ultimate validity for group living species has not yet been tested and will require further simulation analysis (M. G. Efford, pers. comm.). The stable nature of gorilla groups and nonindependence of individuals impacted on our analysis and inflated the variance. However, our SECR gorilla abundance estimates were similar to those based on other methods (Table 3), and we therefore believe that combining SECR and camera trapping is still a very useful approach for gorillas.

### Group membership and social structure

Our results highlight the potential of camera traps for measuring sociodemographic variables in species with differing patterns of sociality such as group size, age/sex structure, demography, distribution of social groups, intersexual variation in home range size, and territoriality or the presence of resident and nonresident individuals in a population.

Our results fit well with what is known about the social structure of these three species. For example, chimpanzee communities showed some overlap (Herbinger et al. 2001), while there was high overlap between gorilla group home ranges (Arandjelovic et al. 2010) and between female elephant group home ranges (White et al. 1993). Group composition of female elephants suggested that they exhibited the same flexible group fission-fusion as seen in savannah elephants (Archie et al. 2006). Core groups sometimes fused temporarily, although in Loango solitary females were also common and accounted for 37% of all identified females (see Fig. S3). Given that such data were previously unfeasible to collect without direct observations or highly invasive radio collaring (Verlinden and Gavor 1998), these results emphasize the potential of remote camera traps in comprehensive population assessment.

### Application at other sites

Several issues need to be considered when applying the proposed approach at other sites. We found interspecific differences in identification rate (see Appendix S3) and species with less variation in individual characteristics (ear markings, skin pigmentation, hair color, or tusk/horn size) would require more intensive capture to obtain an adequate sample size. Additionally, social structure affected capture probability. Stable group living species (gorillas) had the lowest capture probability resulting from a reduced number of “units” in the study area at any one time and as such more intense camera coverage would be advisable.

Furthermore, habitat use patterns can affect capture probability. In our study, gorillas were unevenly distributed across the study area and rarely captured in the central sector (Fig. 3; Head et al. 2012). It is therefore important to account for such patterns during study design to optimize capture probability. Finally, the number of cameras clearly influenced the precision of the SECR estimates (Table 2), indicating that home range size of subject species should dictate grid design to avoid inflated density estimates where the area covered is too small to contain the entire home ranges of some individuals (Gaston et al. 1999).

In more general terms, more intensive camera coverage will substantially reduce the necessary data collection period. Current advances in camera trap image quality (currently 1920 × 1080HD resolution is available compared to 640 × 480 resolution used in this study) will increase individual identification rate. We required 5 days every 2 weeks to monitor the 45 cameras, but recent improvements in battery life and larger video storage capacity would allow cameras to be checked as infrequently as every 3–6 months. With the new technology available, our study could be easily conducted within less than 10 months.

The study design can be scaled up to larger areas using for instance a hierarchical design with clusters of cameras distributed systematically across a region. Camera installation and maintenance can be combined with other activities such as ranger patrols, habituation, tourism, or phenology studies, further reducing the manpower needed.

### Assumptions of the SECR model and resulting limitations of study

In order to ensure adequate sample size, our data collection period spanned 20 months. However, one assumption of the SECR model is that demographic closure exists within a population. Given the duration of data collection, it is possible that individuals died or that individuals which were <6 years of age at the beginning of the study recruited into the >6 years age class by the end. Comparison with other methods and the validation we carried out on the chimpanzee data set suggests that any potential violation was minimal and did not significantly affect the validity of our results. However, we would recommend that any future study use a shorter data collection period (e.g., 6–8 months) with more intensive camera coverage (e.g., 60–80 cameras) in order to avoid possible violations of the assumptions while ensuring adequate sample size.

In addition, SECR models were initially developed for solitary species with independent home ranges such as big cats, and their validity for group living species which share common ranges is yet to be tested. While our results do not indicate that this may have adversely affected our analysis, it would certainly be beneficial for future studies if SECR models were able to incorporate the possibility of a lack of independence among individual home range centers for group living species.

## Conclusions and Recommendations

Our study emphasizes the suitability of combining SECR modeling with camera traps and complementary methods as useful monitoring tools in conservation management. This approach offers enough precision to continuously monitor temporal population changes and thus help in evaluating the effectiveness of conservation strategies. These results have strong implications for management of protected areas or the rapidly increasing number of concessions in extractive industry that require impact mitigation to minimize negative influences on wildlife.

Our study further suggests that there is potential for monitoring population dynamics (e.g., birth/death rate, reproductive success, interbirth intervals, activity patterns, and age/sex structure) of multiple species at the individual level and in the long term. This article examined three species from a total of 19 mammal species recorded on camera traps in Loango, but the study could have been expanded to include additional species which were individually identifiable such as leopard (*Panthera pardus*), sitatunga (*Tragelaphus spekii*), and forest buffalo (*Syncerus caffer nanus*). Furthermore, recent advances in SECR techniques indicate that uncertainty in individual recognition may no longer pose a limitation to estimating population density of elusive species (Chandler and Royle 2011). Future advances in automated identification software will further enlarge the scope for assessing populations at the community, group, and individual levels (Loos and Pfitzer 2012). The aptitude of untrained individuals in species identification confirms that there is potential for widespread capacity building. It shows that these methods could be applied by local staff members, if properly trained on the ground in conservation priority areas. Projects such as “Snapshot Serengeti” (http://www.snapshotserengeti.org/) also highlight the potential of using “citizen scientists” in species identification from camera trap data. Eventually, the proposed approach may help standardize both population assessments across regions and evaluation of conservation effectiveness that environmental managers and decision makers are urgently in need of.

## Supporting Information

**Appendix S1.** Variation in quantity of camera traps distributed across the sampling grid between April 2009 and November 2010.

**Appendix S2.** Individual identification of chimpanzees, gorillas, and elephants.

**Appendix S3.** Methods and results of interobserver reliability testing.

**Figure S1.** The inferred composition of the different chimpanzee communities, and unknown chimpanzees not assigned to any community.

**Figure S2.** The inferred composition of eight gorilla groups, and gorillas not assigned to any group.

**Figure S3.** The inferred composition of different elephant groups (Groups A–U), and solitary individuals.

**Table S1.** Results of full models (GLMM for elephants and LM for chimpanzees) measuring the two-way interaction between sex and number of observations.

## References

[b1] Arandjelovic M, Head J, Boesch C, Kuehl H, Robbins MM, Maisels F (2010). Effective non-invasive genetic monitoring of multiple wild western gorilla groups. Biol. Conserv.

[b2] Arandjelovic M, Head J, Rabanal LI, Mettke E, Boesch C, Robbins MM (2011). Non-invasive genetic estimation of group number and population size in wild central chimpanzees. PLoS One.

[b3] Archie EA, Moss CJ, Alberts SC (2006). The ties that bind: genetic relatedness predicts the fission and fusion of social groups in wild African elephants. Proc. Biol. Sci.

[b4] Baayen RH (2008). Analyzing linguistic data.

[b5] Barlow J, Taylor BL (2005). Estimates of sperm whale abundance in the northeastern temperate Pacific from a combined acoustic and visual survey. Mar. Mamm. Sci.

[b6] Bates D, Maechler M (2010).

[b7] Blake S, Strindberg S, Boudjan P, Makombo C, Bila-Isia I, Ilambu O (2007). Forest elephant crisis in the Congo Basin. PLoS Biol.

[b8] Blake S, Deem SL, Strindberg S, Maisels F, Momont L, Bila-Isia O (2008). Roadless wilderness area determines forest elephant movements in the Congo Basin. PLoS One.

[b9] Boesch C, Boesch-Achermann H (2000). The chimpanzees of the Tai forest: behavioural ecology and evolution.

[b10] Borchers DL, Efford MG (2008). Spatially explicit maximum likelihood methods for capture-recapture studies. Biometrics.

[b11] Buckland ST, Andersen DR, Burnham KP, Laake JL, Borchers DL, Thomas L (2001). Introduction to distance sampling: estimating abundance of biological populations.

[b12] Campbell G, Kuehl HS, Diarrassouba A, N'Goran PK, Boesch C (2011). Long-term research sites as refugia for threatened and over-harvested species. Biol. Lett.

[b13] Caro TM, Young CR, Cauldwell AE, Brown DDE (2009). Animal breeding systems and big game hunting: models and application. Biol. Conserv.

[b14] Chandler RB, Royle JA (2011). Spatially-explicit models for inference about density in unmarked populations. Ann. Appl. Stat.

[b15] Chapman CA, Wrangham RW (1993). Range use of the forest chimpanzees of Kibale: implications for the understanding of chimpanzee social organization. Am. J. Primatol.

[b16] Cipolletta C (2003). Ranging patterns of a western gorilla group during habituation to humans in the Dzanga-Ndoki National Park, Central African Republic. Int. J. Primatol.

[b17] Cohen J (1960). A coefficient of agreement for nominal scales. Educ. Psychol. Meas.

[b18] Dobson AJ (2002). An introduction to generalized linear models.

[b19] Efford MG (2012). http://cran.r-project.org/web/packages/secr/secr.pdf.

[b20] Efford MG, Borchers DL, Byrom AE, Thompson DL, Cooch EG, Conroy MJ (2009). Density estimation by spatially explicit capture-recapture: likelihood-based methods. Modeling demographic processes in marked populations.

[b21] Eggert LS, Eggert JA, Woodruff DS (2003). Estimating population sizes for elusive animals: the forest elephants of Kakum National Park, Ghana. Mol. Ecol.

[b22] Fossey D (1983). Gorillas in the mist.

[b23] Gardner B, Reppucci J, Lucherini M, Royle JA (2010). Spatially explicit inference for open populations: estimating demographic parameters from camera-trap studies. Ecology.

[b24] Gaston KJ, Blackburn TM, Gregory RD (1999). Does variation in census area confound density comparisons?. J. Appl. Ecol.

[b25] Goodall J (1988). In the shadow of man.

[b26] Goswami VR, Lauretta MV, Madhusudan MD, Karanth KU (2011). Optimizing individual identification and survey effort for photographic capture-recapture sampling of species with temporally variable morphological traits. Anim. Conserv.

[b27] Harmsen BJ, Foster RJ, Silver SC, Ostro LET, Doncaster CP (2011). Jaguar and puma activity patterns in relation to their main prey. Mamm. Biol.

[b28] Head JS, Boesch C, Makaga L, Robbins MM (2011). Sympatric chimpanzees (*Pan troglodytes troglodytes*) and gorillas (*Gorilla gorilla gorilla*) in Loango National Park, Gabon: dietary composition, seasonality and inter-site comparisons. Int. J. Primatol.

[b29] Head JS, Robbins MM, Mundry R, Makaga L, Boesch C (2012). Remote video camera traps measure habitat use and competitive exclusion among sympatric chimpanzees, gorillas and elephants in Loango National Park, Gabon. J. Trop. Ecol.

[b30] Herbinger I, Boesch C, Ruthe H (2001). Territory characteristics among three neighboring chimpanzee communities in the Tai National Park, Cote d'Ivoire. Int. J. Primatol.

[b31] Junker J, Blake S, Boesch C, Campbell G, du Toit L, Duvall C (2012). Recent decline in suitable environmental conditions for African great apes. Divers. Distrib.

[b32] Karanth KU, Nichols JD (1998). Estimation of tiger densities in India using photographic captures and recaptures. Ecology.

[b33] Kindberg J, Ericsson G, Swenson JE (2009). Monitoring rare or elusive large mammals using effort-corrected voluntary observers. Biol. Conserv.

[b34] Kuehl HS, Todd A, Boesch C, Walsh PD (2007). Manipulating decay time for efficient large-mammal density estimation: gorillas and dung height. Ecol. Appl.

[b35] Kühl HS, Maisels F, Ancrenaz M, Williamson EA (2008). Best practice guidelines for surveys and monitoring of great ape populations.

[b36] Leuchtenberger C, Ribas C, Magnusson W, Mourão G (2012). To each his own taste: latrines of the giant otter as a food resource for vertebrates in Southern Pantanal, Brazil. Stud. Neotrop. Fauna Environ.

[b37] Loos A, Pfitzer M (2012). Towards automated visual identification of primates using face recognition. http://edas.info/web/iwssip2012/program.html.

[b38] Marques FFC, Buckland ST, Goffin D, Dixon CE, Borchers DL, Mayle BA (2001). Estimating deer abundance from line transect surveys of dung: sika deer in southern Scotland. J. Appl. Ecol.

[b39] Miller CR, Joyce P, Waits LP (2005). A new method for estimating the size of small populations from genetic mark–recapture data. Mol. Ecol.

[b40] Morgan D, Sanz C, Onononga JR, Strindberg S (2006). Ape abundance and habitat use in the Goualougo Triangle, Republic of Congo. Int. J. Primatol.

[b41] Nichols JD, Williams BK (2006). Monitoring for conservation. Trends Ecol. Evol.

[b42] Noss AJ, Gardner B, Maffei L, Cuéllar E, Montaño R, Romero-Muñoz A (2012). Comparison of density estimation methods for mammal populations with camera traps in the Kaa-lya del Gran Chaco landscape. Anim. Conserv.

[b43] Obbard ME, Howe EJ, Kyle CJ (2010). Empirical comparison of density estimators for large carnivores. J. Appl. Ecol.

[b44] O'Connell AF, Nichols JD, Karanth KU (2011). Camera traps in animal ecology.

[b45] Oleaga A, Casais R, Balseiro A, Espí A, Llaneza L, Hartasánchez A (2011). New techniques for an old disease: sarcoptic mange in the Iberian wolf. Vet. Parasitol.

[b46] Otis DL, Burnham KP, White GC, Anderson DR (1978). Statistical inference from capture data on closed animal populations. Wildlife Monogr.

[b47] Plumptre AJ (2000). Monitoring mammal populations with line transect techniques in African forests. J. Appl. Ecol.

[b101] R Development Core Team (2012). R: a language and environment for statistical computing, reference index version 2.1.50. R Foundation for Statistical Computing, Vienna, Austria. ISBN 3-900051-07-0. http://www.R-project.org.

[b48] Robbins MM, Sicotte P, Stewart KJ (2001). Mountain gorillas: three decades of research at Karisoke.

[b49] Robbins MM, Bermejo M, Cipolletta C, Magliocca F, Parnell RJ, Stokes E (2004). Social structure and life history patterns in western gorillas (*Gorilla gorilla gorilla*. Am. J. Primatol.

[b50] Robbins MM, Gray M, Fawcett KA, Nutter FB, Uwingeli P, Mburanumwe I (2011). Extreme conservation leads to recovery of the Virunga mountain gorillas. PLoS One.

[b51] Rodriguez JP, Rodriguez-Clarke KM, Oliveira-Miranda MA, Good T, Grajal A (2006). Professional capacity building: the missing agenda in conservation priority setting. Conserv. Biol.

[b52] Royle JA, Nichols JD, Karanth KU, Gopalaswamy AM (2009). A hierarchical model for estimating density in camera-trap studies. J. Appl. Ecol.

[b53] Siegel S, Castellan NJ (1988). Nonparametric statistics for the behavioural sciences.

[b54] Sollmann R, Malzoni Furtado M, Gardner B, Hofer H, Jácomo ATA, Mundim Tôrres N (2011). Improving density estimates for elusive carnivores: accounting for sex-specific detection and movements using spatial capture-recapture models for jaguars in central Brazil. Biol. Conserv.

[b55] Tranquilli S, Abedi-Lartey M, Amsini F, Arranz L, Asamoah A, Babafemi O (2012). Lack of conservation effort rapidly increases African great ape extinction risk. Conserv. Lett.

[b56] Verlinden A, Gavor IKN (1998). Satellite tracking of elephants in northern Botswana. Afr. J. Ecol.

[b57] White GC, Burnham KP (1999). Program MARK: survival estimation from populations of marked animals. Bird Study.

[b58] White LJT, Tutin CEG, Fernandez M (1993). Group composition and diet of forest elephants, *Loxodonta Africana cyclotis* Matschie 1900, in the Lopé Reserve, Gabon. Afr. J. Ecol.

[b59] Wrege PH, Rowland ED, Thompson BG, Batruch N (2010). Use of acoustic tools to reveal otherwise cryptic responses of forest elephants to oil exploration. Conserv. Biol.

