# Supplementary material for: Effective sociodemographic population assessment of elusive species in ecology and conservation management
Source: Ecol Evol. 2013 Jul 30;3(9):2903–16. doi: 10.1002/ece3.670 (PMC3790539; doi:10.1002/ece3.670)
Supplement: Supplementary file 1 [file ece30003-2903-SD1.doc]

**Appendix S1.** Variation in the number of remote video camera traps distributed across the sampling grid between April 2009 and November 2010. Camera trap models used included Scoutguard 550 <http://www.scoutguard.com/>, Bushnell Trophy Cam <http://www.bushnell.com/> and Sony handycam <http://www.sony.co.uk/hub/handycam-camcorders> with trailmaster 700V <http://www.trailmaster.com/>.

**Period Number of cameras**

April 2009 – June 2009 8

July 2009 – August 2009 12

September 2009 – October 2009 18

November 2009 – January 2010 24

February 2010 – August 2010 45

September 2010 – November 2010 34

**Appendix S2.** Photos illustrating the individual identification of chimpanzees, gorillas and elephants from camera traps in Loango NP, Gabon.

A1 B1 C1 D1 E1 F1

**
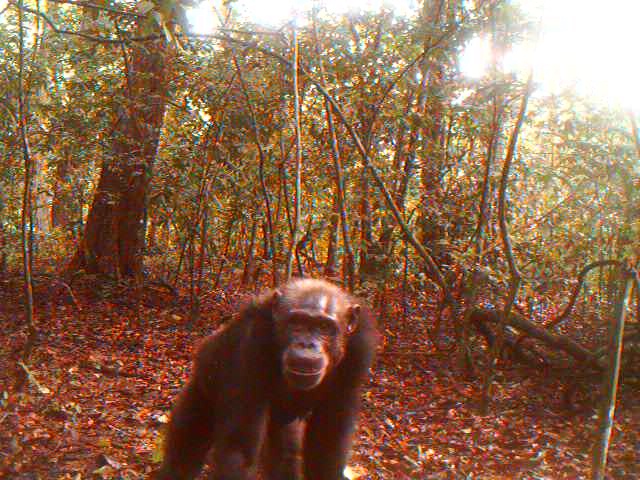

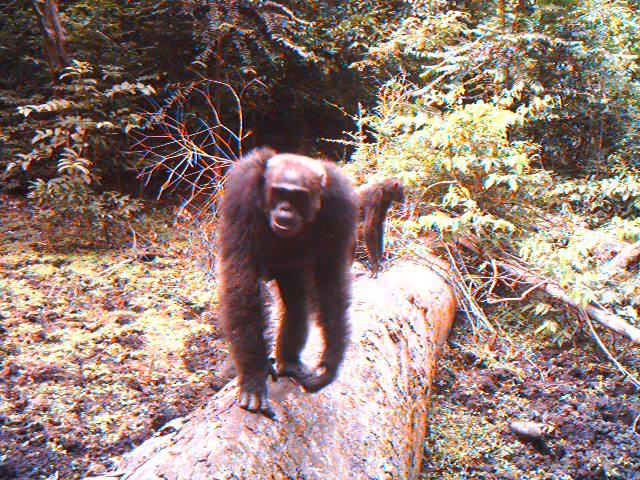

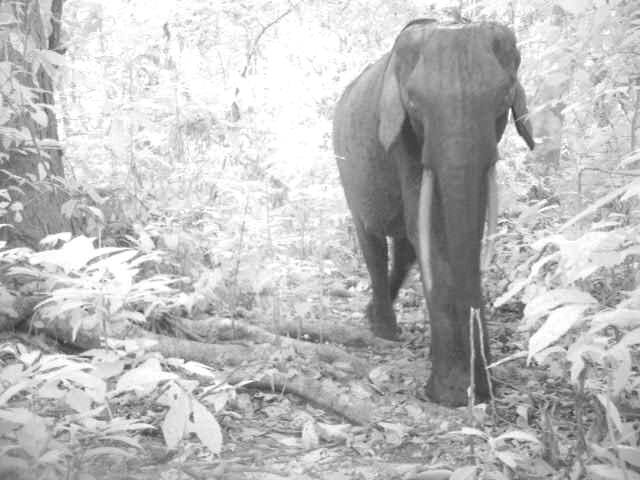

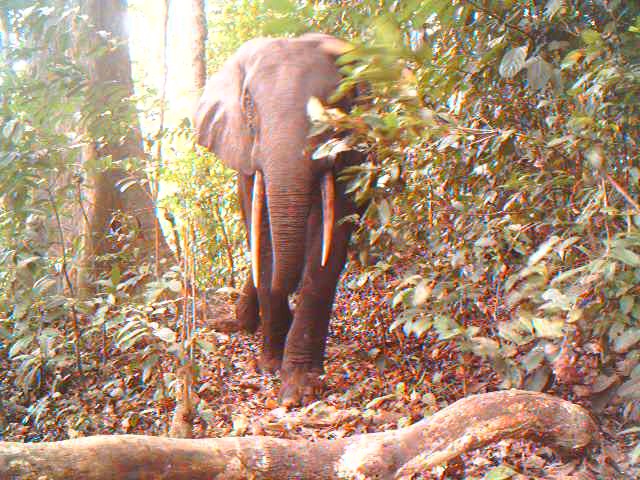

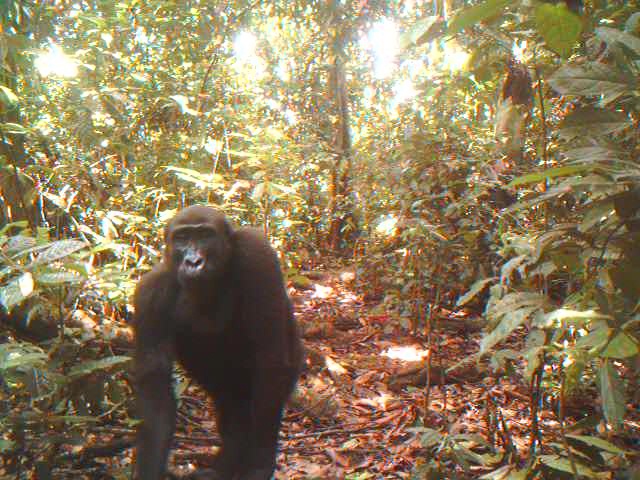

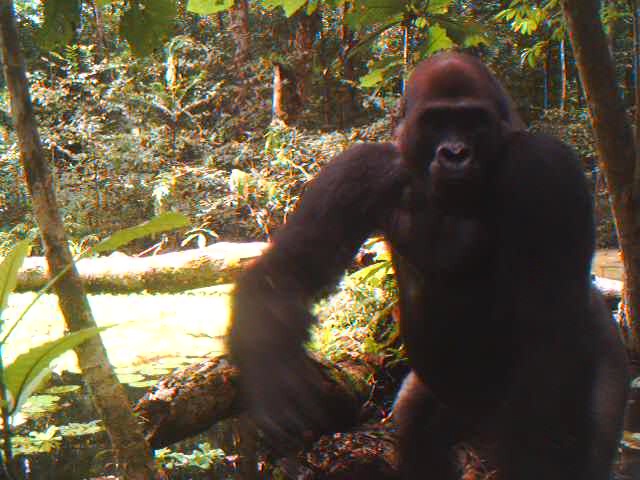
**

A2 B2 C2 D2 E2 F2

**
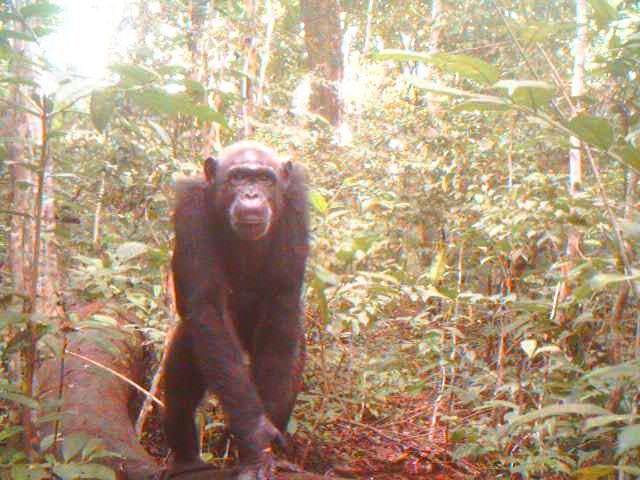

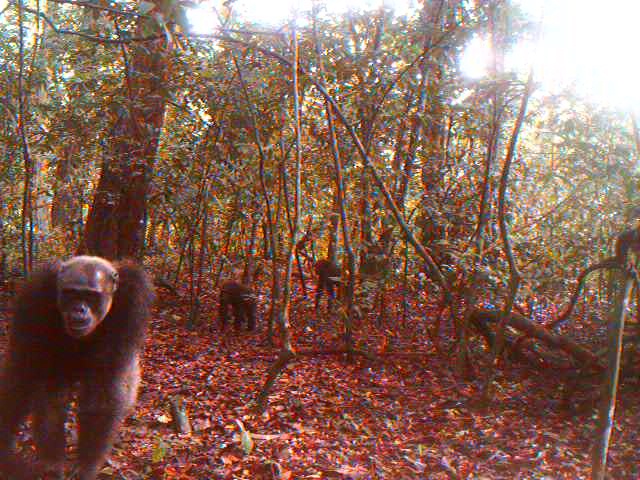

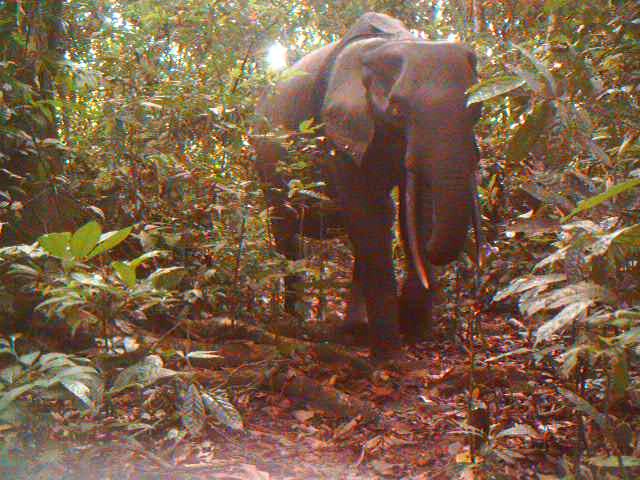

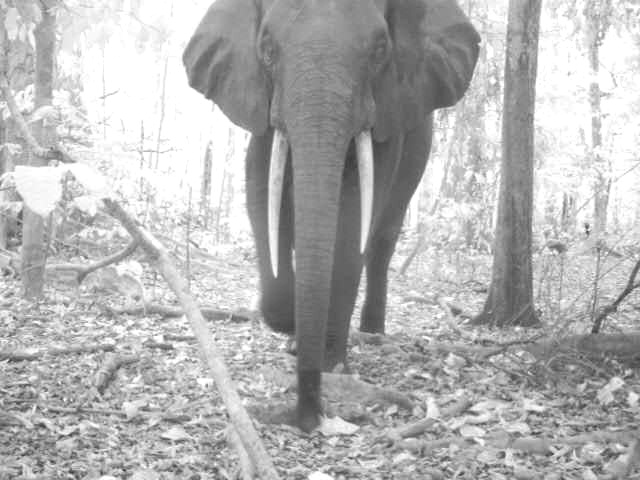

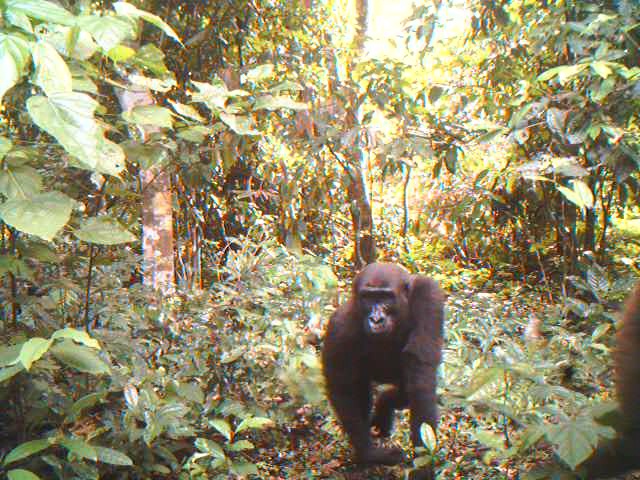

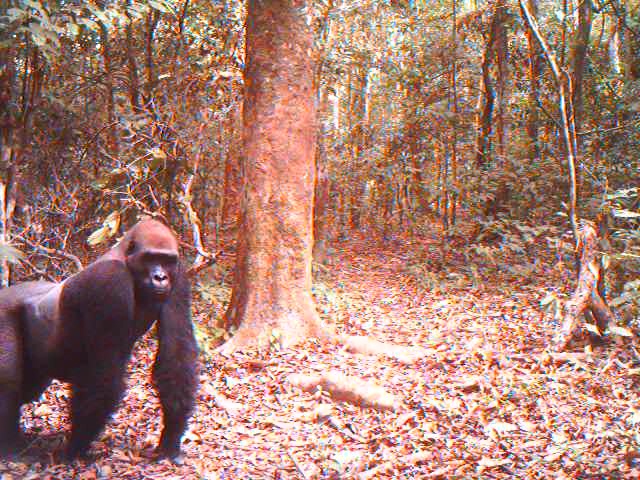
**

A1 & A2 = adult female chimpanzee ‘Roxy’; B1 & B2 = adult male chimpanzee ‘Jacketman’; C1 &C2 = adult female elephant ‘Elsie’; D1 & D2 = adult male elephant ‘Sid’; E1 & E2 = adult female gorilla ‘Spoony’; F1 & F2 = adult male gorilla ‘Brett’.

**Appendix S3.** Methods and results of inter-observer reliability testing to confirm the reliability of the identification method.

**Methods:** Participants studied independent pairs of video images (N=40 pairs of chimpanzees and elephants, N=24 pairs of gorillas) with accompanying still shot photos of their subject species. Potential pairs were of the same sex and approximate age, and videos with similar image and subject quality were used together. Participants were shown equal numbers of male and female pairs in each test and there were equal numbers of pairs and non-pairs as judged by the principal observer. No video or photo was used more than once in the test and there was no time restriction for observers. Inter-observer reliability was measured using Cohen’s Kappa (*K*) coefficient (Cohen 1960): $K=\frac{\Pr(a)-\Pr(e)}{1-\Pr(e)}$

where Pr(*a*) is the relative observed agreement among raters, and Pr(*e*) the hypothetical probability of chance agreement. Positive values indicate agreement was higher than that expected by chance. Magnitude of agreement was measured according to the guidelines suggested by Landis & Koch (1977) where agreement ranging between 0-0.2 = slight, 0.21-0.4 = fair, 0.41-0.6 = moderate, 0.61-0.8 = substantial, and 0.81-1 = almost perfect.

**Results:** Table showing results of inter-observer reliability test (Cohen’s Kappa) per species and participant category. Numbers refer to agreement between participants and the principal observer (JH) where 1.0 = perfect agreement. Figures in brackets and italics display the *P* value of the agreement. “Mean agreement” refers to mean agreement score across all species/sex categories for each participant category.

Chimpanzee Chimpanzee Gorilla Gorilla Elephant Elephant **Mean**

Males Females Males Females Males Females **Agreement**

Experienced observer 1 0.8 1.0 1.0 0.66 0.9 1.0 **0.89**

*(<0.001) (<0.001) (<0.001) (0.02) (<0.001) (<0.001)*

Experienced observer 2 0.9 0.9 0.67 0.82 1.0 1.0 **0.88**

*(<0.001) (<0.001) (0.01) (0.004) (<0.001) (<0.001)*

Ape project field assistant 1 0.7 0.8 0.83 0.27 1.0 1.0 **0.77**

*(0.002) (<0.001) (0.003) (0.3) (<0.001) (<0.001)*

Ape project field assistant 2 0.7 0.5 0.83 0.64 0.9 1.0 **0.76**

*(0.002) (0.02) (0.003) (0.02) (<0.001) (<0.001)*

Inexperienced observer 1 0.7 0.7 0.66 0.65 0.8 0.9 **0.74**

*(0.002) (0.001) (0.02) (0.02) (<0.001) (<0.001)*

National park ecoguard 1 0.5 0.7 0.5 0.47 0.9 1.0 **0.68**

*(0.03) (0.002) (0.08) (0.1) (<0.001) (<0.001)*

Inexperienced observer 2 0.4 0.5 0.67 0.64 0.7 1.0 **0.65**

*(0.07) (0.02) (0.02) (0.02) (0.002) (<0.001)*

National park ecoguard 2 0.8 0.5 0.33 0.06 0.7 1.0 **0.57**

*(<0.001) (0.02) (0.2) (0.8) (0.002) (<0.001)*

**Mean across all categories 0.7 0.7 0.7 0.5 0.9 1.0**

**References**

Cohen, J. (1960) A coefficient of agreement for nominal scales. *Educational and Psyhological Measurement,* **20,** 37-46.

Landis, J.R. & Koch, G.G. (1977) The measurement of observer agreement for categorical data. *Biometrics,* **33,** 159-174.
