# Supplementary material for: Effective sociodemographic population assessment of elusive species in ecology and conservation management
Source: Ecol Evol. 2013 Jul 30;3(9):2903–16. doi: 10.1002/ece3.670 (PMC3790539; doi:10.1002/ece3.670)
Supplement: Supplementary file 2 [file ece30003-2903-SD2.doc]

**Figure S1.** The inferred composition of four chimpanzee communities ‘BT’, ‘R’, ‘Y’, ‘BB’, and “unknown” chimpanzees not assigned to any community.

**A=Adult, J=Juvenile, M=Male, F=Female, *= female has an infant, °= female has a juvenile.** **The ‘Y’ community is actually made up of 6 sub-groups (dotted lines) not observed at the same time but observed in the same locations, and based on their location and chimpanzee social structure it is geographically improbable that they belong to different communities. Similarly, there were two adult females which were attributed to the ‘BB’ community based on their geographic location.**

**Figure S2.** The inferred composition of 8 gorilla groups: ‘Indegho’, ‘Southern’, ‘Coastal’, ‘Brett’, ‘Atananga’, ‘Big Ears’, ‘Faro’ and ‘Northern’; and gorillas not assigned to any group.

**A=Adult, BB=Black back, SA=Sub adult, J=Juvenile, I=Infant, M=Male, F=Female, U=unknown, *= female has an infant, °= female has a juvenile. Because western gorilla groups are considered to be stable (Robbins *et al*. 2004), in addition to the identified adult and sub-adult individuals we inferred the minimum group size and composition of each group without identifying each individual through determination of age/sex class of all individuals seen during one video trigger. Therefore,** **where “I.D code” cells are blank but age and sex cells filled in, the individual was never individually identified on the cameras, but group composition and age/sex class was inferred from video footage of the entire group where other individuals were successfully identified (therefore “unassigned individual” females and sub-adults may be from identified groups and represent non-i.ded individuals within groups).**

**Figure S3.** The inferred composition of different elephant groups (Groups A – U), and solitary individuals.

**A=Adult, AD=Adolescent, M=Male, F=Female,*= female has an infant, °= female has a juvenile, ?*?= infant died during the study. Dotted arrows refer to male/female dyads observed together on at least one occasion, and males found together on at least one occasion are in the same box. Females were considered solitary when they were not accompanied by another adult or adolescent female, but they could be accompanied by dependent offspring.**
