# Supplementary material for: Effective sociodemographic population assessment of elusive species in ecology and conservation management
Source: Ecol Evol. 2013 Jul 30;3(9):2903–16. doi: 10.1002/ece3.670 (PMC3790539; doi:10.1002/ece3.670)
Supplement: Supplementary file 3 [file ece30003-2903-SD3.doc]

**Table S1.** Results of full models (GLMM for elephants and LM for chimpanzees) measuring the two-way interaction between sex and number of observations. Interactions are denoted by :.

***GLMM Elephants:***

**Estimate Std. t MCMC HPD95 HPD95 *P*MCMC**

**Error value mean lower upper**

**(Intercept)** -0.327 0.178 -1.834 -0.289 -0.619 0.024 *

**Sex** 0.078 0.265 0.295 0.038 -0.481 0.533 *

**No. of obs** 0.521 0.188 2.774 0.533 0.158 0.879 *

**Sex : No of obs** 0.642 0.257 2.498 0.633 0.110 1.120 **0.013**

****not shown because it has no meaningful interpretation***

***LM Chimpanzees:***

**Estimate Std. Error t value *P* value**

**(Intercept)** 0.772 0.183 *

**Sex** 0.464 0.403 *

**No. of obs** 1.418 0.176 *

**Sex : No of obs** -0.594 0.449 -1.322 0.196

****not shown because it has no meaningful interpretation***
